# Experimental Study of Macro and Microgeometric Defects in Drilled Carbon Fiber Reinforced Plastics by Laser Beam Machining

**DOI:** 10.3390/ma11081466

**Published:** 2018-08-18

**Authors:** Pedro F. Mayuet Ares, Juan Manuel Vázquez Martínez, Mariano Marcos Bárcena, Antonio J. Gámez

**Affiliations:** Department of Mechanical Engineering & Industrial Design, Faculty of Engineering, University of Cadiz, Av. Universidad de Cádiz 10, E-11519 Puerto Real (Cadiz), Spain; juanmanuel.vazquez@uca.es (J.M.V.M.); mariano.marcos@uca.es (M.M.B.); antoniojuan.gamez@uca.es (A.J.G.)

**Keywords:** laser beam machining, carbon fiber reinforced polymer, hole quality characteristics, geometrical parameters, roughness parameters, taper, heat affected zone

## Abstract

Plastic matrix composite materials are an excellent choice for structural applications where high strength-weight and stiffness-weight ratios are required. These materials are being increasingly used in diverse industrial sectors, particularly in aerospace. Due to the strict tolerances required, they are usually machined with drilling cycles due to the type of mounting through rivets. In this sense, laser beam drilling is presented as an alternative to conventional drilling due to the absence of tool wear, cutting forces, or vibrations during the cutting process. However, the process carries with it other problems that compromise the integrity of the material. One of these is caused by the high temperatures generated during the interaction between the laser and the material. In this work, variance analysis is used to study the influence of scanning speed and frequency on macro geometric parameters, surface quality, and defects (taper and heat affected zone). Also, in order to identify problems in the wall of the drill, stereoscopic optical microscopy (SOM) and scanning electron microscopy (SEM) techniques are used. This experimental procedure reveals the conditions that minimize deviations, defects, and damage in machining holes.

## 1. Introduction

Reinforced plastic and polymers were originally designed for improving the physicochemical properties and reducing the weight of the classic materials, mainly metallic alloys, commonly used in different industrial sectors. Particularly among them, carbon fiber reinforced polymers (CFRP) are widely applied in sectors that require lightweight materials, the ability to withstand great efforts, high stiffness, and excellent conditions for fatigue [1]. These characteristics, especially those related to their low density, make CFRP materials highly attractive for industrial applications in a great diversity of sectors, such as the aerospace or automotive sectors. However, changes in global economics and politics have led to a production standstill of CFRP, even a decrease in certain sectors, due to large cuts, mainly on the defense sector, although this effect has been diminished by the increase of consumption for commercial applications. Therefore, some producers expect an increase in demand of about 75% in the industrial sector (excluding the aerospace) by 2020, specifically in energy, transport, and civil engineering [1].

In the aeronautical industry, the use of non-metal matrix composites (NMMC) has increased sharply in the last few years as they are becoming more and more used in the building of structural elements of commercial and military airships. In these cases, organic matrix-based composite materials reinforced with high-strength fiber or particles are used [2,3]. Among them, CFRP is one of the most widely used. These composite materials are characterized by their high compositional heterogeneity and anisotropy, low thermal conductivity, matrix sensitivity at high temperatures, and the abrasive nature of the fibers used in their manufacture [4,5,6]. These features complicate the manufacturing processes of the airship elements based in CFRPs.

Moreover, most of the CFRP elements used in the airship’s building need to be machined by drilling or contour milling. However, the aforementioned CFRP features negatively affect the machinability of these elements when traditional or conventional machining processes are used. The material heterogeneity and the abrasive behavior of carbon fiber conditions negatively affect the tool life [7,8,9,10,11,12]. For this reason, the material and geometry of the tools must be adapted to the machining conditions required by the material in order to reduce the tool wear and the thermal and mechanical defects produced during the cutting process to maintain the functional performance of the manufacturing process [13]. Moreover, tool life is one of the most relevant parameters that condition the economic performance of the process. However, currently, the optimization of the cutting tools used in the machining processes of CFRP work pieces is considered a difficult task [14]. In this sense, different non-traditional machining processes can be thought to be profitable and economical alternatives to solve the troubles previously mentioned. Therefore, alternatives to this type of machining are becoming more common, such as abrasive water jet (AWJM), electrical discharge machining (EDM), ultrasonic machining, or laser beam machining (LBM) [12,15,16,17,18,19].

**Figure 1:**
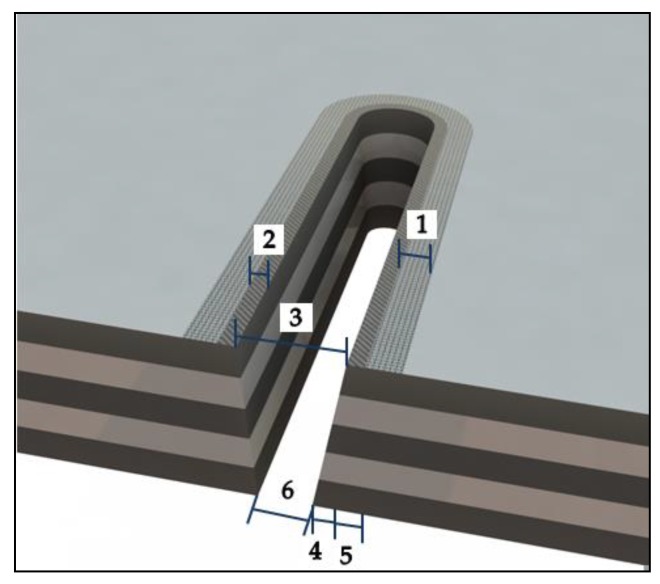
Typical defects on the specified surface zones of the material by laser in polymer matrix composites. At the beam entrance, 1. heat affected matrix, 2. matrix recession, and 3. kerf width. At the exit: 4. matrix recession, 5. heat affected matrix, and 6. kerf width.

In particular, LBM possesses several advantages. For example, it is a technology without contact and without abrasive particles, therefore eliminating contact forces, tool wear, and vibrations during cutting. In addition, laser cutting can be easily automated and performed at high speed [20,21,22,23]. On the other hand, the challenges for the laser to machining the carbon fiber are the minimization or elimination of the thermal damage due to the great difference between the mechanical and thermal properties of the material constituents and to maintain a high cutting speed [24,25,26,27,28,29]. Therefore, defects, such as the heat affected zone (HAZ), the carbonization of the fiber, the removal or loss of resin, or the delamination due to intense thermal effects, are the main obstacles for industrial applications of carbon fiber machining by laser, as seen in Figure 1.

In this paper, carbon fiber reinforced polymer (CFRP) is machined by a fiber laser doped with ytterbium. In order to analyze the quality of the cut, the influence of the cutting parameters has been analyzed. Specifically, a number of tests varying the scanning speed (S) and frequency (Fr) have been performed to obtain holes. The test pieces have been inspected by combining stereoscopic optical microscopy (SOM) and scanning electron microscopy (SEM) techniques. Furthermore, macro and micro-geometric parameters, such as diameter (D), cylindricity (C), straightness (St), roundness (R-In/R-Out), and roughness (Ra), have been studied. Finally, the quality of the holes has been evaluated by measuring defects such as the heat affected zone (HAZ) or the taper angle (T). 

## 2. Materials and Methods 

### 2.1. Experimental Procedure

Table 1 includes the main features of the CFRP pieces (80 mm × 80 mm × 5.85 mm) used in this study. Holes of 7.92 mm diameter, by combining circular trajectories using a fiber laser doped with ytterbium (Yb), have been laser drilled. This diameter is commonly used for assembling structural elements in aeronautical industry. 

The influence of pulsed frequency, F, and scanning speed, S, on the thermal caused defects has been analyzed [2,21,22,30]. Thus, Table 2 includes the selected values in this study. The rest of parameters have remained constant during the tests, Table 3. Before them, trials with each set of parameters were carried out to determine the thickness of the material in each pass. 

For the realization of the drills, a strategy of marking by shading was chosen. To program it, Laser Mark software (version 2.3.0) was used. In addition, it is important to mention that each consecutive pass was performed by modifying the scanning direction by 45°. In this way, the heat damage distribution is homogenized among all the fiber directions observed in the stacking sequence.

To distinguish the most significant parameters for evaluation results, analysis of variance (ANOVA) for a 95% confidence interval was employed. Thus, the F value and the p value have been analyzed to measure the evidence against the null hypothesis. After that, contour charts for each variable studied in the experimental (D, D_In_, D_Out_, St, C, Ra, T and HAZ) were obtained. 

### 2.2. Macro and Micro-Geometrical Evaluation

The diameter at the inlet and outlet of the material were evaluated using a digital profile projector Tesa Visio 300 (Hexagon, Stockholm, Sweden).

To analyze the macro and micro geometric deviations, replicas of the holes were fabricated due to the impossibility of direct measuring on the material. These replicas were made with a polymer type F80 Ra (R.G.X, Plastiform, Madrid, Spain) with the ability to guarantee stability during the measurement process for diameters greater than 4 mm. In this way, roundness was calculated at the entrance and exit of the drill (R-In/R-Out), along with straightness (St), cylindricity (C), and roughness (Ra). 

**Figure 2:**
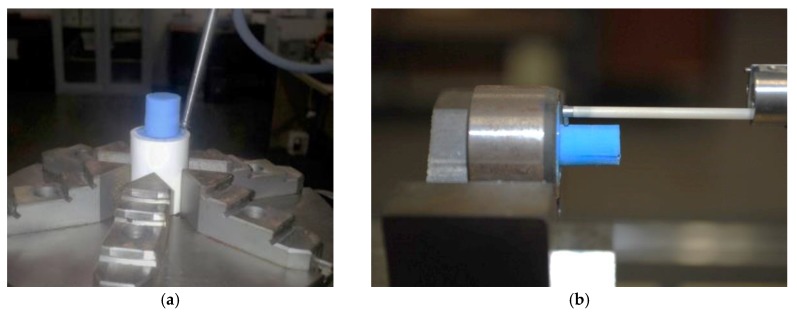
Measurements made on the replicas, (**a**) example of measurement of macro geometry; and (**b**) example of measurement of micro-geometry.

A station of measurement Mahr MMQ44 Form Tester (Mahr, Göttingen, Germany) was used to measure the roundness at the entrance and exit of the drill, the cylindricity of the entire profile of the drill, and the straightness in four separate generatrices to 90°, as shown in Figure 2a. On the other hand, the evaluation of the micro-geometric defects was carried out using a Mahr Perthometer Concept PGK120 (Mahr, Göttingen, Germany) equipment to obtain four 2-D separated profiles, as seen in Figure 2b. For that, a stylus with a 2 μm tip radius and 90° tip angle was used for the measurements, where the surface finishing parameter employed to indicate the surface quality was the arithmetic mean roughness. 

### 2.3. SOM/SEM Evaluation

The test pieces were inspected by microscopy techniques using Tesa Visio 300 (Hexagon, Stockholm, Sweden). Subsequently, to calculate the taper (T) with the inlet diameter (D_In_), the outlet diameter (D_Out_), and the thickness of the material (t), ImageJ software (1.50i) was used. Also, to obtain a thermal damage ratio on the surface of the material, the damaged diameter (D_d_) was also calculated.

(1) $$\theta =\tan^{-1}\left(\frac{D_{\mathrm{In}}+D_{\mathrm{out}}}{2t}\right)$$

(2) $$\mathrm{HAZ}=\frac{D_{d}}{D_{\mathrm{In}}}$$

Finally, to complete the inspection of the defects, scanning electron microscopy Quanta 200 (SEM, Thermo Fisher Scientific, Waltham, MA, USA) was used. 

## 3. Results and Discussion

### 3.1. Results of the Analysis

Table 4 and Table 5 show the data obtained for each test and the ANOVA analysis, respectively.

### 3.2. Analysis of Macro and Micro-Geometrical Deviations

#### 3.2.1. Diameter Evaluation

**Figure 3:**
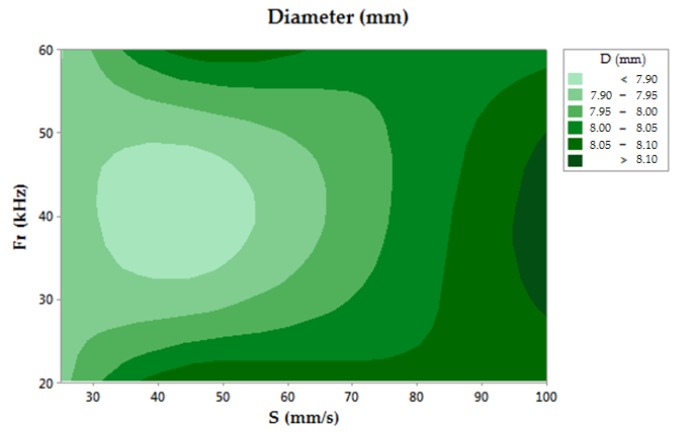
Contour chart of the diameter.

The nominal diameter of the drilled hole was 7.92 mm. The results obtained from the diameter measurements are shown in Figure 3. In this contour chart, the closest theoretical value was obtained for low scanning speed and medium frequency level conditions. Under these conditions, 7.929 mm was the best result, obtained in test number 2. In contrast, measurement values over 8 mm in diameter were detected for S = 100 mm/s. The analysis of the results shows that a low scanning speed involves more accuracy for diameter dimensions of the drilled holes. On the other hand, the ANOVA analysis was not conclusive for rejecting the null hypothesis. However, a higher F-value was shown by S, suggesting a greater relation between this parameter with respect frequency. 

#### 3.2.2. Roundness Evaluation

**Figure 4:**
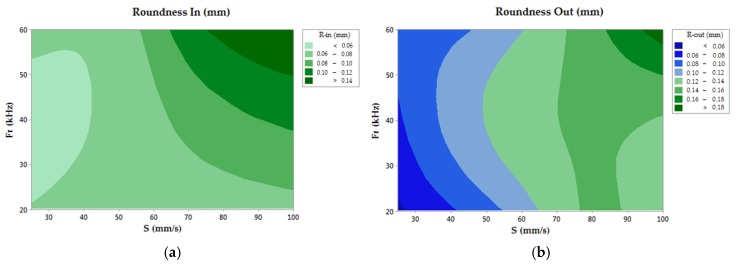
(**a**) Contour chart of the inlet roundness; (**b**) Contour chart of the outlet roundness.

In the case of roundness deviations, the ANOVA analysis showed a higher influence of S, allowing for the rejection of the null hypothesis for the values obtained in the measurement of the drilled hole. Additionally, a tendency to increase the roundness deviations could be observed for high values of the studied process parameters (Fr and S), as seen in Figure 4.

Generally speaking, R-In increased Fr and S, although the minimum was found for low sweep speeds and medium frequency values. R-Out showed a similar trend, but the lowest roundness deviations were measured for the lowest frequency and scanning speed.

**Figure 5:**
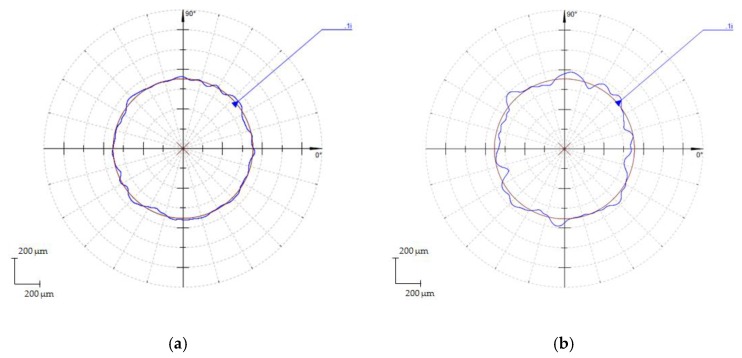
Roundness profile, (**a**) Hole inlet, Test 12. (S = 100 mm/s and Fr = 60 kHz); (**b**) Hole Outlet, Test 12. (S = 100 mm/s and Fr = 60 kHz).

Also, relevant differences could be detected between the entrance and exit sections of the holes, presenting an important growth in the deviation values for the exit section with respect to the entrance, as seen in Figure 5. This behavior was specially related to the increase in the number of laser scanning stages and the focal length variation between stages.

#### 3.2.3. Straightness Evaluation

**Figure 6:**
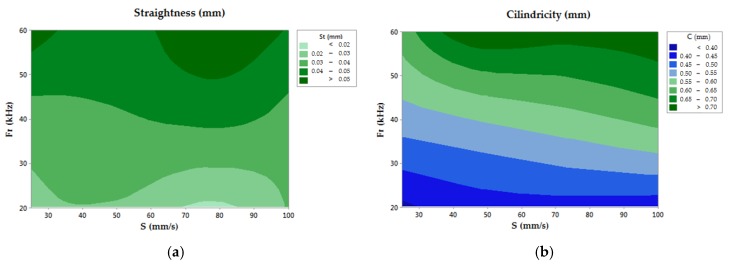
(**a**) Contour chart of the straightness; (**b**) Contour chart of the cylindricity.

In the case of straightness, the null hypothesis could be rejected using the frequency parameter. Regarding the scanning speed, the straightness behavior did not indicate a significant tendency, showing similar behavior for all the S values of the range studied. In contrast, an increasing trend have been followed in the straightness deviations as a function of frequency, as can be seen in Figure 6a. Under these considerations, a maximum value of 0.057 mm is reported for the Fr = 60 kHz tests. The evaluation of the measured straightness behavior allowed for the confirmation of a direct dependency with the energy of the pulse, improving the hole features for higher frequency conditions.

#### 3.2.4. Cylindricity Evaluation

**Figure 7:**
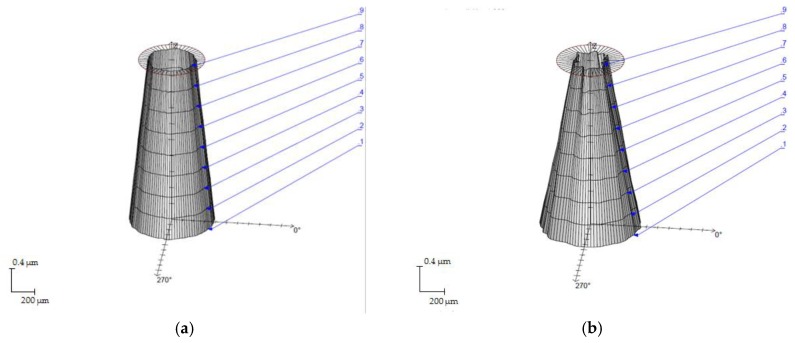
Cylindricity profile, (**a**) Hole inlet, Test 1. (S = 20 mm/s and Fr = 20 kHz); (**b**) Hole outlet, Test 12. (S = 100 mm/s and Fr = 60 kHz).

Cylindricity deviations were affected by the same considerations as the straightness parameter, showing a relevant increasing trend as a function of frequency, and obtaining a maximum value of 0.729 mm. In this aspect, a slighter influence from the scanning speed is reported, as can be seen in Figure 6b. This behavior may confirm that the Fr parameter could be used reject the null hypothesis. On the other hand, the cylindricity allowed for the calculation of a first approximation of the taper angle, being directly related as shown in Table 4. Thus, Figure 7 shows the cylindricity profiles in tests with opposite parameters showing the taper of the drills.

#### 3.2.5. Surface Finish Evaluation

**Figure 8:**
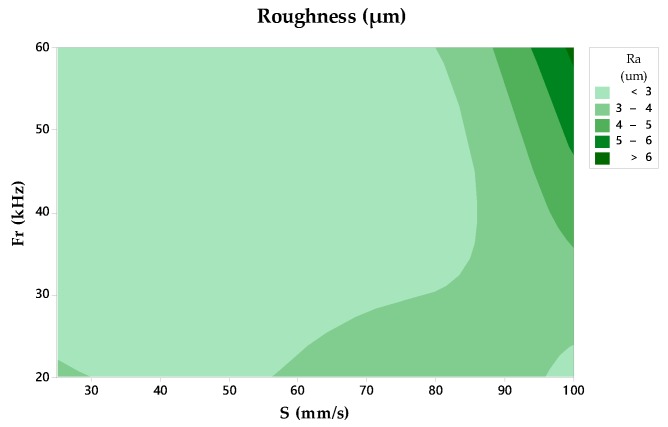
Contour chart of the roughness.

The average roughness parameter Ra was used to assess the surface finish. It was measured by constructing replicas of the drilled holes. In this evaluation, the dependency between the studied parameters could not be verified, where the F value of S shows a higher value. However, the highest Ra values were found for the highest scanning speeds and frequency conditions [29], as seen in Figure 8. Values of Ra between 2.20 µm and 6.22 µm are reported.

### 3.3. Damage and Defects Analysis on the Drilled Holes

Table 5 shows that the p-value may vary as a function of the analyzed response variable (T, HAZ). First, for taper growth, experimental data confirmed the null hypothesis for the Fr parameter, showing a direct influence in the defect formation process. On the other hand, the HAZ allowed for the rejection of the null hypothesis for the different process parameters studied.

#### 3.3.1. Taper Angle

**Figure 9:**
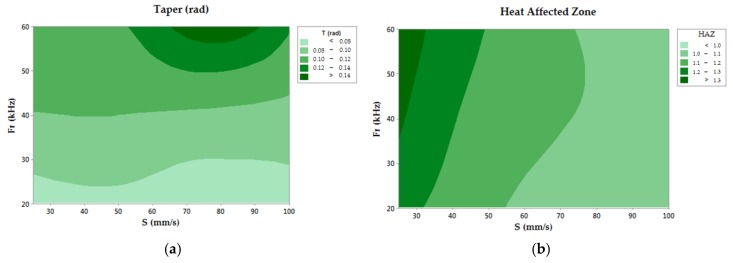
(**a**) Contour chart of the taper angle; (**b**) Contour chart of the heat affected zone.

**Figure 10:**
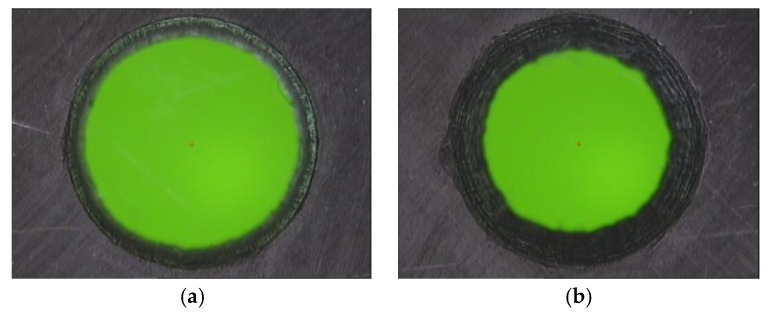
Effect of the frequency, (**a**) Test 7. (S = 75 mm/s and Fr = 20 kHz); (**b**) Test 9. (S = 75 mm/s and Fr = 60 kHz).

Results obtained from the ANOVA analysis revealed the relevance of Fr in the taper formation, as seen in Figure 9a. In this way, taper formation became critical for higher values of Fr, resulting in a maximum value of 0.152 in test number 9. This behavior may have caused an increase in the conicity of the drill hole as a function of the pulse energy of the laser beam. However, a direct dependency between S and taper defect was not detected, where similar values are reported for all of the studied speed range. Under these conditions, Fr may be considered the most influential parameter in the process of taper development [30,31,32], according to Figure 10.

#### 3.3.2. Heat Affected Zone

**Figure 11:**
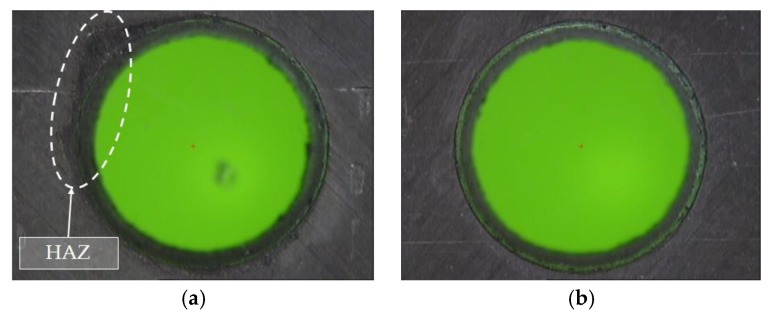
Effect of scanning speed, (**a**) Absence of reinforcement and matrix at the entrance of the drill, Test 1. (S = 20 mm/s and Fr = 20 kHz); (**b**) Hole without defects, Test 10. (S = 100 mm/s and Fr = 20 kHz).

Based on the evaluation of the HAZ, defects caused by heat damages allowed for the rejection of the null hypothesis. However, its relevant to remark that the F-value showed a higher influence of S than Fr. In Figure 10b, the taper decreased when the scanning speed increased. This was due to the fact that, with high S, the laser beam affected the composite surface for a shorter time, leading to a decrease in the HAZ as expected [33]. Additionally, in Figure 11, the defect caused by heat is shown in detail, disappearing when higher speeds were used. In addition, it can be seen how the defect appeared only at the border of the drill, which favors the extension of the zone due to the conductivity of the reinforcement in the longitudinal direction of the fiber.

### 3.4. SEM Analysis

The heat affected zone is considered the most important defect in the machining of CFRP. SEM analysis allows for more detailed observation distinguishing some typical defects produced in the material [34,35,36].

**Figure 12:**
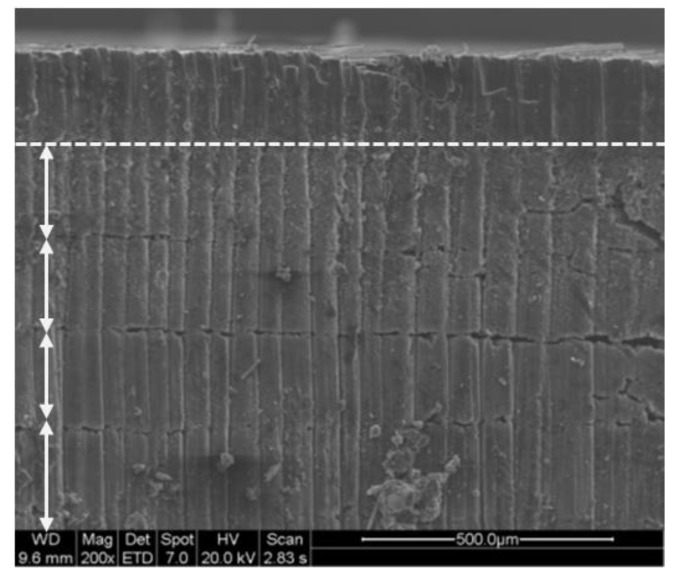
Clearance distance per pass in test 1 (S = 20 mm/s and Fr = 20 kHz).

**Figure 13:**
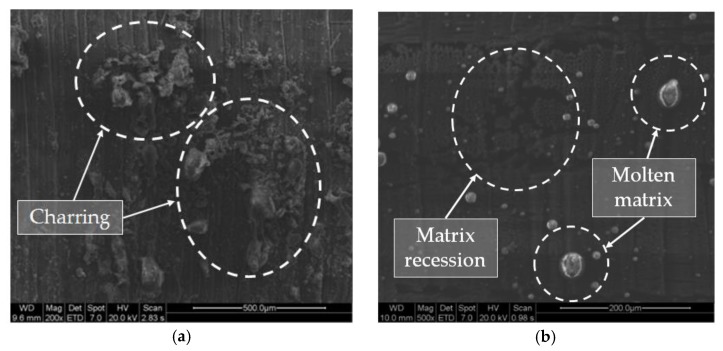
(**a**) Burned fiber debris deposited on the drill wall (charring); (**b**) Absence of resin due to the evaporation process. Detail of molten matrix adhered to the drill wall.

In Figure 12, it is possible to appreciate what has been described in the experimental procedure related to the number of stages necessary to eliminate material-producing defects similar to delamination [33]. In addition, charring at the drill wall has been observed, basically caused by the high temperatures reached during cutting, as seen in Figure 13a. On the other hand, it has also been possible to identify areas of material with matrix recession. This occurs when the matrix and fibers are removed at different rates owing to their different thermo-physical properties [27,37,38], as seen in Figure 13b. In addition, molten matrix deposited on the wall of the drill was also detected.

## 5. Conclusions

An experimental study was conducted to determine the influence of process parameters on hole geometry and kerf wall characteristics in laser beam drilling machining of CFRP composites. Based on this, the following conclusions can be made:

- The roundness measurements obtained at the inlet and outlet of the drill reveal that smaller dimensional deviations were obtained when selecting low scanning speeds and pulse frequencies. In addition, it was found that the deviation of the roundness at the exit is always greater (increasing in some tests up to 250%), mainly due to the influence of the focal distance.
- Pulsed frequency and scanning speed also affected straightness and cylindricity in a great depth, especially for the latter. The straightness deviation decreased with frequency and the deviation of cylindricity presented higher values relating to the taper defect, especially when the pulsed frequency was increased.
- The roughness of the drilled holes did not seem to have a significant relationship with the parameters evaluated. However, reduced scanning speeds had better surface quality values. Specifically, for S = 20 mm/s, measurements of about 2 μm were obtained. Roughness values were considered low compared to those obtained through other machining processes.
- The taper angle was closely related to the frequency and affected by the speed of scanning, where an increase in the energy of the pulse decreased the appearance of the defect. The minimum value of the angle was obtained in trial 1 with 0.069 rad. However, this drill presented high damage caused by temperature.
- This study showed the direct influence of the parameters proposed in the experiment. However, the scanning speed determined the appearance of defects in the surface of the material. In this study, no damage was recorded when selecting high speeds and low pulse frequencies (S = 100 mm/s and Fr = 20 kHz).
- Similar to the evaluation of the surface finish, the influence of the cutting parameters in the evaluation of the diameter could not be clearly stated with this test. However, the selection of reduced scanning speeds seemed to offer a higher dimensional accuracy of the hole.
- SEM analysis detected characteristic defects associated with the laser machining of composites on the hole’s surface. Thus, charring localization, absence of matrix, and deposition of matrix remains on it was recorded. These defects occurred due to the high temperatures produced during the process.

## Figures and Tables

**Table 1:** CFRP pieces features.

Type of Material	Composition	Production Method	Technical Specification
Layers of unidirectional carbon fiber with epoxy resin matrix and a symmetrical stacking sequence of (0/90/45/-45/45/-45)	Intermediate module fiber (66%) and epoxy resin (34%)	Pre-preg and autoclaved at 458° ± 5° at a pressure of 0.69 MPa	AIMS-05-01-XXX

**Table 2:** Cutting parameters selected for the tests.

Parameter	Levels			
S (mm/s)	25	50	75	100
Fr (kHz)	20	40	60	

**Table 3:** Constant parameters during the tests.

Power (W)	Wavelength (nm)	Spot Diameter (µm)	Working Mode	Scanning Distribution	Atmosphere
10	1062	60	Pulsed	Shaded	Environment

**Table 4:** Results of the evaluated variables.

Test	S (mm/s)	Fr (kHz)	D (mm)	D_In_ (mm)	D_Out_ (mm)	St (mm)	C (mm)	Ra (µm)	T (rad)	HAZ
1	25	20	7.934	0.041	0.057	0.028	0.388	3.15	0.069	1.233
2	25	40	7.929	0.035	0.077	0.036	0.524	2.20	0.099	1.314
3	25	60	7.896	0.045	0.084	0.056	0.622	2.38	0.118	1.355
4	50	20	8.103	0.046	0.092	0.029	0.430	2.84	0.074	1.119
5	50	40	7.878	0.043	0.121	0.039	0.556	2.39	0.100	1.160
6	50	60	8.081	0.045	0.105	0.045	0.722	2.80	0.118	1.194
7	75	20	8.066	0.046	0.138	0.018	0.429	3.78	0.070	1.031
8	75	40	7.996	0.055	0.142	0.042	0.583	2.50	0.097	1.096
9	75	60	8.024	0.070	0.144	0.057	0.719	2.76	0.152	1.097
10	100	20	8.048	0.047	0.123	0.031	0.418	2.68	0.070	1.000 ^1^
11	100	40	8.127	0.062	0.138	0.037	0.616	4.37	0.094	1.049
12	100	60	8.032	0.079	0.192	0.049	0.729	6.22	0.122	1.049

^1^ Without a heat affected zone.

**Table 5:** ANOVA analysis of the evaluated variables.

S			Fr		
Parameter	F-value	*p*-value	Parameter	F-value	*p*-value
D	2.19	0.190	D	0.55	0.602
R-In	4.99	0.045	R-In	3.60	0.094
R-Out	12.80	0.005	R-Out	2.79	0.139
St	0.08	0.970	St	18.02	0.003
C	5.70	0.034	C	128.86	0.000
Ra	0.83	0.229	Ra	0.41	0.686
T	0.83	0.524	T	32.32	0.001
HAZ	143.19	0.000	HAZ	22.61	0.002
